# Regulating Protein Immobilization During Cell‐Free Protein Synthesis in Hyaluronan Microgels

**DOI:** 10.1002/adbi.202400668

**Published:** 2025-02-17

**Authors:** Anika Kaufmann, Kateryna Ivanova, Julian Thiele

**Affiliations:** ^1^ Leibniz‐Institut für Polymerforschung Dresden e. V. Hohe Straße 6 01069 Dresden Germany; ^2^ Institute of Chemistry Otto von Guericke University Magdeburg Universitätsplatz 2 39106 Magdeburg Germany

**Keywords:** bifunctional microgels, cell‐free protein synthesis, GFP, protein immobilization

## Abstract

Cell‐like platforms are being studied intensively for their application in synthetic biology to mimic aspects of life in an artificial environment. Here, micrometer‐sized, bifunctional microgels are used as an experimental platform to investigate the interplay of cell‐free protein synthesis (CFPS) and in situ protein accumulation inside the microgel volume. In detail, microgels made of hyaluronic acid (HA) are first modified with different amounts of nitrilotriacetic acid (NTA) moieties to characterize the capability and maximum capacity of binding His‐tag modified GFP. CFPS is optimized for the system used here, particularly when using a linear DNA template. Afterward, HA‐microgels are functionalized with the linear DNA template and Ni^2+^‐activated NTA moieties to bind in situ synthesized GFP‐His. CFPS and parallel protein accumulation within the microgels are observed over time to determine the GFP‐His binding to the microgel platform. With this approach, the study presents the first steps for a platform to study the temporal‐spatial regulation of protein synthesis by tailored protein binding or release from the microgel matrix‐based reaction environment.

## Graphical abstract

Bifunctional microgels carrying a linear DNA template and Ni^2+^‐activated NTA moieties are used as platform for cell‐free protein synthesis and in situ protein immobilization. By varying the concentration of NTA moieties in the microgels, the amount of GFP‐His immobilized inside the microgel and released to the microgel environment can be regulated.

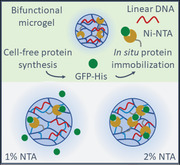

## 1 Introduction

Cell‐free protein synthesis (CFPS) has been widely employed in fundamental and applied biology for over 60 years.^[^ ^1^, ^2^ ^]^ With CFPS, it is possible to focus all the supplemented energy on the production of the protein of interest as it is not necessary to sustain cell viability and growth, avoiding the metabolic and cytotoxic burdens.^[^ ^3^, ^4^ ^]^ That way, proteins that are difficult to synthesize since they are toxic to host cells in vivo, as well as membrane proteins or those with modified or unnatural amino acids, can be synthesized.^[^ ^5^ ^]^ Due to their broad versatility, scalability, and portability,^[^ ^6^ ^]^ CFPS systems have been adopted in metabolic engineering and synthetic biology applications. Different platforms were studied for CFPS systems where the protein synthesis machinery is derived from a cell lysate which can be generated from prokaryotic and eukaryotic organisms,^[^ ^4^, ^5^ ^]^ e.g., *E. coli*, which is still the most‐used and versatile system,^[^ ^7^ ^]^ but also plant extracts as wheat germ^[^ ^8^ ^]^ or tobacco BY‐2,^[^ ^9^ ^]^ and mammalian cells.^[^ ^10^ ^]^ The lysate can be extracted by a French press,^[^ ^11^, ^12^ ^]^ sonication,^[^ ^13^, ^14^ ^]^ or bead beating.^[^ ^15^, ^16^ ^]^

Cell‐free technologies are primarily performed in bulk solutions, thus not reflecting living cells, characterized by phenomena such as macromolecular crowding that influences biochemical processes, for example, protein folding, their interactions, and subcellular organization.^[^ ^17^, ^18^ ^]^ Progressively, the influence of a crowding environment on cellular organization was discovered in both eukaryotes and prokaryotes.^[^ ^19^, ^20^ ^]^ Many researchers have shown that CFPS can not only be performed in bulk reactions at the milliliter scale but can also be scaled down to pico‐ and femtoliter scales in encapsulated cell‐like systems^[^ ^7^ ^]^ such as liposomes,^[^ ^21^, ^22^ ^]^ polymersomes,^[^ ^23^, ^24^ ^]^ proteinosomes,^[^ ^25^ ^]^ vesicles,^[^ ^26^ ^]^ coacervates,^[^ ^27^ ^]^ droplets,^[^ ^28^ ^]^ or microgels.^[^ ^29^, ^30^, ^31^, ^32^ ^]^ The insights that were gained from these encapsulated systems are crucial for the development of synthetic cells, e.g., understanding the dynamics of complex enzymatic reactions in highly crowded small volumes,^[^ ^28^ ^]^ CFPS of actin‐like structural proteins within polymersomes to mimic aspects of the *E. coli* cytoskeleton,^[^ ^24^ ^]^ in vitro synthesis of an integral membrane protein in polymer membranes as a strategy to generate membrane‐like assemblies,^[^ ^23^ ^]^ or creation of sub‐compartments.^[^ ^25^ ^]^ Crowding also influences CFPS; for example, coacervation creates an artificial cell‐like environment with a significantly increased rate of mRNA production, a two orders of magnitude larger binding constant between DNA and T7 RNA polymerase, and a five to six times larger rate constant for transcription in crowded environments.^[^ ^27^ ^]^

Microgels with nitrilotriacetic acid (NTA) moieties were used to immobilize recombinant poly histidine‐tagged (His‐Tag) proteins^[^ ^30^, ^31^ ^]^ through the already known and widely used Ni‐mediated His‐Tag method,^[^ ^33^ ^]^ allowing for spatial regulation of protein synthesis. In addition to coupled NTA moieties, bifunctional microgels also contain linear DNA to locally combine transcription, translation, and immobilization of the synthesized protein while offering the possibility of adjusting their composition, for example, by screening different concentrations of the materials involved. The use of bifunctional microgels for CFPS was expanded to synthesizing and immobilizing a functional enzyme that converts malonate to malonyl CoA.^[^ ^32^ ^]^ However, the concentration of Ni^2+^‐activated moieties coupled to microgels was kept constant so far. For this reason, we focus on investigating bifunctional microgels with varying NTA moieties content to expand their future usage in macromolecular crowding and confinement. The effect of crowding on interactions and kinetics of CFPS directly impacts the understanding of biochemical networks in vivo.^[^ ^27^ ^]^ Often polymers such as polyethylene glycol (PEG), dextran, Ficoll, and poly(sodium 4‐styrenesulfonate) (PSS), or proteins were used, and the influence of crowder size is discussed.^[^ ^34^ ^]^ Here, we present the first steps for regulating the release or binding of in situ synthesized proteins to study temporal‐spatial regulation of protein synthesis. With this approach, a basic protocol can be established to tailor the macromolecular crowding in the microgels’ surrounding environment by dynamically synthesizing proteins as crowding agents.

## 2 Results and Discussion

### 2.1 Formation of Microgels

**Figure 1:**
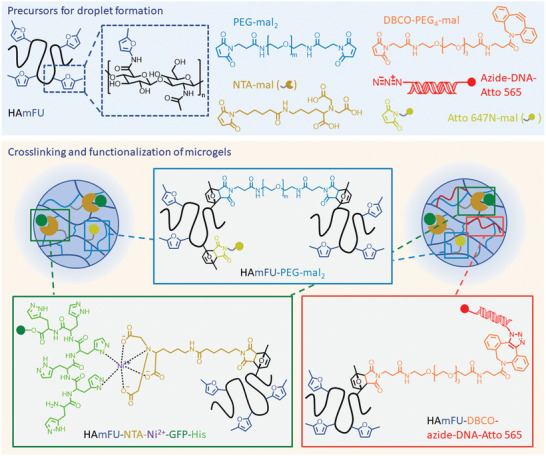
Structure of precursor molecules and scheme of HAmFU and HAmFU‐DBCO microgel crosslinking as well as immobilization of His‐tagged GFP‐His to Ni^2+^‐activated NTA‐modified microgels and functionalization of HAmFU‐DBCO microgels with azide‐DNA‐Atto 565 for in situ synthesis of GFP‐His.

Polymer microgels were produced via droplet microfluidics by loading water‐in‐oil microemulsions containing the precursor molecules (**Figure** **1**) into microfluidic devices (cf. Figure S1, Supporting Information). 5‐methylfuran‐modified hyaluronic acid (HAmFU) was prefunctionalized with NTA‐maleimide (NTA‐mal) for a later Ni^2+^‐activation and GFP‐His binding (Figure 1). The crosslinker PEG‐dimaleimide (PEG‐mal_2_) was added in a separate aqueous phase to avoid crosslinking in solution before droplet formation (cf. Figure S1a, Supporting Information). For HAmFU prefunctionalized with dibenzocyclooctyne‐PEG_4_‐maleimide (DBCO), DNA and NTA‐mal (HAmFU‐DBCO‐azide‐DNA‐Atto 565, Figure 1), a premixing with PEG‐mal_2_ was necessary and the mixture was directly injected into the microfluidic device (cf. Figure S1b, Supporting Information). It was shown before that a mixing of both phases inside the microfluidic device led to inhomogeneous droplet formation, while the premixing allowed for homogeneous droplet formation.^[^ ^31^ ^]^ After droplet formation both types of microgels, HAmFU and HAmFU‐DBCO, were formed by thermal crosslinking with PEG‐mal_2_ (Figure 1).

In a first step, HAmFU was functionalized with different amounts of NTA‐mal (NTA) ranging from 1% (w/v) to 4% (w/v) to determine how much NTA could be bound and how this affects the GFP‐His binding. The size of the formed emulsion droplets varied from 22.7 ± 1.9 µm for those containing 4% NTA to 26.5 ± 2.0 µm for those with 2% NTA (**Table** **1**). The corresponding microgel size was 30.4 ± 1.6 µm for microgels with 1% NTA, 35.7 ± 3.0 µm for microgels with 2% NTA, and 35.2 ± 1.3 µm for microgels with 3% NTA. The 5‐methylfuran groups of HAmFU serve as binding sites for functionalization with NTA moieties as well as for crosslinking with PEG‐mal_2_. For that, HAmFU is preincubated with NTA to ensure maximized functionalization separated from the crosslinking step with PEG‐mal_2_. Thus, if the number of NTA moieties increases, fewer binding sites are available for crosslinking, resulting in a less dense hydrogel network. The amount of hydrogel precursors was kept constant at 15.4 µmol and 0.4 µmol for 3.5% (w/v) HAmFU and 1% (w/v) PEG‐mal_2_, respectively, in all experiments, while the amount of NTA was varied from 4.8 µmol for 1% NTA to 9.7 µmol for 2% NTA to 14.5 µmol for 3% NTA, and 19.4 µmol for 4% NTA. Based on these calculations, 3% NTA represents the upper limit for modification of the HAmFU hydrogel precursor. This was confirmed experimentally by the incorporation of 4% NTA into the hydrogel network. While the formation of emulsion droplets was still possible, no solidification and microgel formation occurred due to a lack of sufficient binding sites available for further crosslinking. Furthermore, the microgels’ degree of swelling (DS) is influenced by their composition, especially the crosslinker density.^[^ ^31^, ^35^, ^36^ ^]^ The DS is calculated by the diameter of the emulsion droplet (d (droplet) and the corresponding microgel diameter (d (microgel)) after crosslinking.

(1) $$\mathrm{DS}\;=\frac{d\;\left(\mathrm{microgel}\right)-d\left(\mathrm{droplet}\right)}{d\;\left(\mathrm{droplet}\right)}\times 100$$

**Table 1:** Diameter of emulsion droplets and HAmFU‐microgels crosslinked with PEG‐mal_2_ and functionalized with different amounts of NTA‐mal (n = 50 ± s.d.).

NTA‐mal [% (w/v)]	Emulsion droplet diameter [µm]	Microgel diameter [µm]	DS [%]
1	25.1 ± 1.7	30.4 ± 1.6	21.0 ± 10.9
2	26.5 ± 2.0	35.7 ± 3.0	34.5 ± 15.3
3	24.3 ± 2.3	35.2 ± 1.3	44.5 ± 14.7
4	22.7 ± 1.9	‐	‐

Two‐sample t‐test of DS, where ** *p* < 0.01, **** *p* < 0.0001;

1% NTA/2% NTA ****, 2% NTA/3% NTA **, 1% NTA/3% NTA ****.

Here, microgels with 1% NTA had the smallest DS with 21.0 ± 10.9%, which is significantly lower than the DS for microgels with 2% NTA, which was 34.5 ± 15.3%. The DS for microgels with 3% NTA was 44.5 ± 14.7, which is significantly higher as the DS for microgels with 2% NTA and 1% NTA (Table 1). Thus, the higher availability of 5‐methylfuran groups resulted in a denser network for microgels with 1% NTA, while microgels with 3% NTA yielded a hydrogel network with fewer crosslinks.

### 2.2 Immobilization of GFP‐His to HAmFU‐Microgels

**Figure 2:**
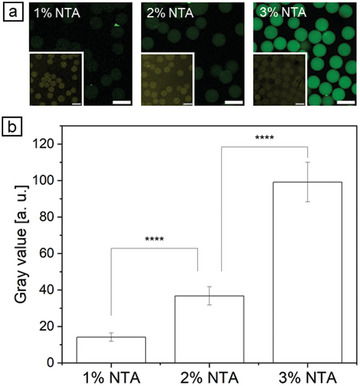
Binding of commercial GFP‐His (25 ng µL^−1^ in TRIS buffer) to HAmFU‐microgels presenting different amounts of Ni^2+^‐activated NTA groups. a) CLSM images of bound GFP‐His to microgels after washing non‐bound GFP‐His, inset showing microgels stained with Atto 647N (scale bar indicates 50 µm). b) Calculated gray values, background was subtracted (n = 50 ± s.d.). Two‐sample *t*‐test, where **** *p* < 0.0001.

GFP‐His was used as a model protein to investigate the availability of different amounts of NTA moieties in the hydrogel network. By activating these NTA moieties with Ni^2+^‐ions, a specific and reversible binding with the His‐Tag of any synthesized protein could be achieved. It has previously been shown that microgels without NTA moieties or without Ni^2+^‐activation of these moieties cannot bind GFP‐His and that GFP without His‐Tag was not immobilized to Ni^2+^‐activated NTA‐modified microgels.^[^ ^31^ ^]^ Depending on the microgels’ average mesh size and diffusivity, unbound protein freely diffuses out of the microgel. In the presence of Ni^2+^‐activated NTA‐groups presented by the microgels, confocal laser scanning microscopy (CLSM) images showed that different NTA concentrations within the microgels affected the binding of GFP‐His (**Figure** **2**a). In detail, for microgels containing 1% NTA, the gray value corresponded to 14.2 ± 2.3. With increasing amounts of NTA moieties presented by the microgels, the gray value also increased to 36.8 ± 5.0 for microgels with 2% NTA and to 99.2 ± 10.9 for microgels with 3% NTA (Figure 2b). This confirms that the higher amounts of NTA moieties are also available for protein immobilization via His‐Tag.

### 2.3 CFPS in Solution

The experimental design for CFPS includes selection of the CFPS system, choice of DNA sequence and design of the DNA template, selection or design of reagents, and selection or design of the energy mix.^[^ ^37^ ^]^ Here, polymerase chain reaction (PCR) was performed to obtain a linear azide‐modified DNA, which can be coupled to HAmFU‐DBCO‐microgels. This DNA can be used to synthesize the protein GFP‐His, which can bind via its His‐Tag to Ni^2+^‐activated, NTA‐functionalized microgels.^[^ ^31^ ^]^ A fragmented version of GFP (deGFP) was used here, which is more translatable in cell‐free systems than the original eGFP.^[^ ^15^ ^]^

**Figure 3:**
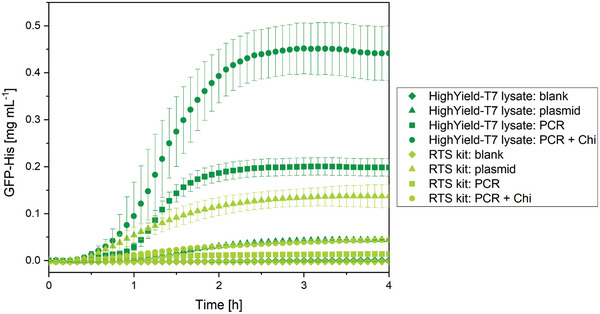
Time‐dependent synthesis of GFP‐His via CFPS in solution at 29 °C using a HighYield‐T7 lysate or RTS kit while applying linear DNA received via PCR (PCR), with additional Chi DNA (PCR + Chi), or plasmid DNA (plasmid) as well as no DNA as control (blank) (n = 3 ± s.d.).

For CFPS, two different approaches based on *E. coli* lysate were investigated to assess the suitability of synthesizing GFP‐His from a linear template. First, a commercial CFPS kit, namely RTS 100 *E. coli* HY kit from biotechrabbit GmbH, was used, which showed a GFP‐His concentration of 0.014 ± 0.001 mg mL^−1^ for CFPS from the linear DNA template after 4 h (**Figure** **3**). The concentration of GFP‐His was increased to 0.044 ± 0.001 mg mL^−1^ when adding 2 µm Chi DNA indicating a threefold higher CFPS rate. The double‐stranded Chi DNA was studied by Marshall and coworkers to avoid the degradation of linear PCR products by RecBCD complexes inside the lysate.^[^ ^38^ ^]^ For comparison, the synthesis of GFP‐His from the plasmid template was performed, where a GFP‐His concentration of 0.137 ± 0.025 mg mL^−1^ was achieved, indicating a threefold higher production rate of GFP‐His from the plasmid template than for the linear template with Chi DNA when using the commercial RTS 100 *E. coli* HY kit under the investigated conditions.

In a second approach, a commercial *E. coli* HighYield‐T7 lysate from Cube Biotech GmbH was used and combined with all necessary reagents for CFPS according to Levine et al.^[^ ^14^ ^]^ With this approach, a GFP‐His concentration of 0.441 ± 0.058 mg mL^−1^ was achieved after 4 h when using the linear DNA template with the addition of Chi DNA, which is ten times higher as for the RTS 100 *E. coli* HY kit. Without Chi DNA, half the GFP‐His synthesis rate was reached with a GFP‐His concentration of 0.198 ± 0.019 mg mL^−1^. With the plasmid template, a GFP‐His concentration of 0.044 ± 0.001 mg mL^−1^ was produced, which is as high as for the RTS 100 *E. coli* HY kit when using the linear template with Chi DNA. Under the selected conditions in this study, the RTS 100 *E. coli* HY kit with a plasmid template performed better, while the approach with a commercial *E. coli* HighYield‐T7 lysate and homemade CFPS reagents achieved a higher GFP‐His concentration with the linear plasmid. Since the reagent composition for the RTS 100 *E. coli* HY kit is not known in detail, further comparison between the approaches is not possible. The typical reaction time for a reporter protein such as eGFP includes an initial phase of 0–60 min when mRNA is synthesized,^[^ ^37^ ^]^ which can also be observed here. A quasilinear accumulation of the produced protein usually appears within 1–8 h,^[^ ^37^ ^]^ and was accomplished here after 2 h. Afterwards, a plateau was reached, which indicated a complete reaction.

### 2.4 Mobility of Ribosomes in HAmFU‐DBCO‐Microgels

To transfer CFPS in solution to CFPS in microgels, it is necessary to ensure that CFPS components can diffuse into HAmFU‐microgels, which are modified with DBCO for a later coupling of DNA. NTA amounts of 1%, 2%, and 3% were pre‐incubated with HAmFU‐DBCO for a later in situ coupling of synthesized GFP‐His to Ni^2+^‐activated NTA moieties. The crosslinker concentration was reduced to 0.5% (w/v) PEG‐mal_2_ to investigate whether higher mobility can be achieved with a less crosslinked microgel network. For an NTA amount of 3% no microgel formation appeared after successful emulsion droplet formation. As discussed above, based on the degree of functionalization it is possible to theoretically calculate the concentration of the materials used. 3.7% (w/v) HAmFU‐DBCO was employed, which corresponds to an amount of substance of 15.2 µmol, while the concentration of 3% (w/v) NTA refers to 14.5 µmol and 0.5% (w/v) PEG‐mal_2_ to 0.2 µmol. Theoretically, the concentrations are suitable, and potentially, microgels could be formed. However, experimentally, this was not achieved. From a practical point of view, PEG‐mal_2_ is added only in the last step, just before being injected into the microflow cell. Therefore, it should not be excluded that since HAmFU‐DBCO was previously incubated with DNA, then NTA and Atto 647N‐mal, the remaining 5‐methylfuran groups were not available for the formation of the microgel network.

**Figure 4:**
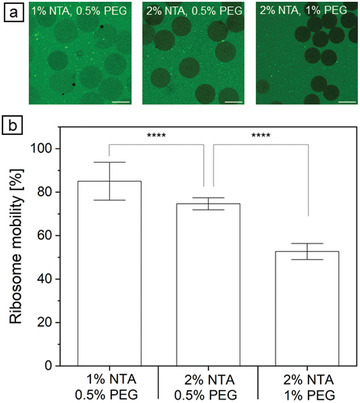
Ribosome mobility in 1% and 2% NTA‐functionalized HAmFU‐DBCO‐microgels with 0.5% and 1% PEG as crosslinker. a) CLSM images of microgels with incubated ribosomes (scale bars indicate 50 µm). b) Calculated ribosome mobility in microgels (n = 50 ± s.d.). Two‐sample *t*‐test, where **** *p* < 0.0001.

For this reason, we determined the mobility of ribosomes in HAmFU‐DBCO‐microgels with 1% NTA and 2% NTA (**Figure** **4**). Since ribosomes have a molecular weight of 2.3 MDa,^[^ ^39^ ^]^ they are among the largest molecules in the CFPS mixture, and their mobility is intended to serve as a model for all other CFPS components. The ribosome mobility was calculated as the quotient of gray values of areas inside and outside the microgels. HAmFU‐DBCO‐microgels with 2% NTA and 1% (w/v) PEG‐mal_2_ as crosslinker showed a significantly lower ribosome mobility with 52.7 ± 3.7% than HAmFU‐DBCO‐microgels with 2% NTA and 0.5% (w/v) PEG‐mal_2_ having a ribosome mobility of 74.6 ± 2.8% (Figure 4b). The ribosome mobility was further increased for HAmFU‐DBCO‐microgels with 1% NTA and 0.5% (w/v) PEG‐mal_2_ to 85.0 ± 8.7%, which is significantly higher as for HAmFU‐DBCO‐microgels with 2% NTA and 0.5% (w/v) PEG‐mal_2_. In summary, the ribosome mobility was decreased when using a higher crosslinker concentration due to a denser microgel network. Furthermore, a higher concentration of NTA moieties decreased the ribosome mobility.

### 2.5 CFPS of GFP‐His and its Immobilization by NTA Moieties in HAmFU‐DBCO‐DNA‐Microgels

After successful CFPS of GFP‐His in solution by comparing different CFPS approaches, CFPS and immobilization of GFP‐His in HAmFU‐DBCO‐microgels with 1% and 2% NTA was investigated. The functionalization with the azide‐modified linear DNA template was realized via strain‐promoted azide‐alkyne cycloaddition to HAmFU‐DBCO. Microgels were fabricated via droplet microfluidics (cf. Figure S1b, Supporting Information) with a reduced amount of 0.5% (w/v) PEG‐mal_2_ as a crosslinker. As shown above, a reduced crosslinker concentration resulted in a less dense microgel network and increased ribosome mobility. Furthermore, it was shown before that higher protein synthesis rates can be achieved when reducing the PEG‐mal_2_ concentration from 1% (w/v) to 0.5% (w/v).^[^ ^31^ ^]^ For HAmFU‐DBCO‐DNA microgels with 1% NTA and 2% NTA, the amount of bound DNA was analyzed as a first step (Figure S2, Supporting Information). The gray value was 18.9 ± 1.3 for microgels with 1% NTA and 12.7 ± 2 for microgels with 2% NTA, respectively (Figure S2b, Supporting Information). Therefore, microgels with 1% and 2% NTA were successfully functionalized with DNA, and further analyzed to examine the GFP‐His immobilization after CFPS.

**Figure 5:**
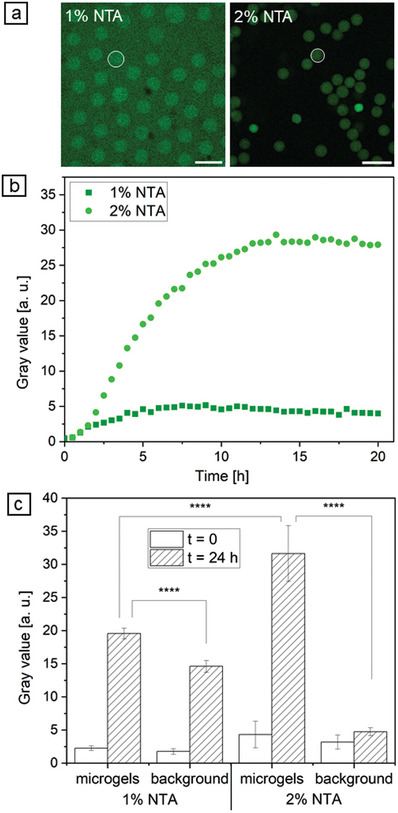
CFPS of GFP‐His in 1% and 2% NTA‐functionalized microgels. a) CLSM images of GFP‐His after 20 h CFPS (scale bars indicate 100 µm). b) Time‐dependent change in the gray value of the marked microgels (white circle in (a)), the background was subtracted. c) Calculated gray values for GFP‐His bound to microgels and found in the background (n = 50 ± s.d.). Two‐sample *t*‐test, where **** *p* < 0.0001.

Successful CFPS of GFP‐His was achieved in both compositions of microgels, with 1% and 2% NTA (**Figure** **5**a). It is possible to observe that Ni^2+^‐activated NTA moieties presented by microgels were able to immobilize the in situ synthesized GFP‐His as the fluorescence intensity within the microgels is higher than in the background. Nonetheless, for microgels with 1% NTA, the gray values in the background increased in parallel to the gray values inside the microgels as determined by a time‐dependent change in gray value for one representative microgel (Figure S3a, Supporting Information). A plateau of synthesized and bound GFP‐His was reached after ≈5 h in the microgels and the background. For microgels with 2% NTA, the gray values in the background were nearly constant over time and substantially lower. While gray values in the microgels achieved a maximum after ≈12 h, a saturation is obtained markedly later than for microgels with 1% NTA (Figure S3b, Supporting Information). Subtracting the gray values of the background from the gray values in the microgels gave an overview of specific GFP‐His binding to Ni^2+^‐activated NTA‐functionalized microgels (Figure 5b). Here, it is clearly visible that microgels with 2% NTA exhibit higher GFP‐His binding. For microgels with 1% NTA, the NTA concentration was insufficient to bind all GFP‐His, which was synthesized, resulting in a diffusion of GFP‐His outside the microgel. The mean gray value of the background increased for microgels with 1% NTA from initially 1.8 ± 0.4 to 14.6 ± 0.9 after 20 h, while inside these microgels, the gray values increased from 2.2 ± 0.3 to 19.6 ± 0.8 after 20 h (Figure 3c).

Therefore, microgels with 1% NTA exhibit 1.3 times higher gray values after 20 h compared to the background demonstrating its binding capacity for GFP‐His. For microgels with 2% NTA, the gray values ascended inside the microgels from initially 4.3 ± 2.0 to 31.6 ± 4.2 after 20 h. Here, the gray values in the background nearly did not change over time, starting with 3.2 ± 1.1 to 4.7 ± 0.6 after 20 h (Figure 5c). In summary, microgels with 2% NTA had a 6.7 times higher gray value after 20 h compared to the background, clearly indicating its higher ability to bind in situ synthesized GFP‐His. Since the background remained nearly constant, it could be assumed that these microgels bound the synthesized GFP‐His quantitatively. Considering the higher ribosome mobility in HAmFU‐DBCO‐microgels with 1% NTA (cf. Figure 4), a higher GFP‐His synthesis rate during CFPS might be possible compared to HAmFU‐DBCO‐microgels with 2% NTA. In fact, when comparing the accumulated gray values of GFP‐His in microgels and background after 4 h, a higher gray value of 37.6 was achieved for HAmFU‐DBCO‐microgels with 1% NTA (cf. Figure S3a, Supporting Information) than for HAmFU‐DBCO‐microgels with 2% NTA (Figure S3b, Supporting Information), where 20.8 was reached. This is an indication of a higher GFP‐His production rate of HAmFU‐DBCO‐microgels with 1% NTA due to their less dense microgel network and higher mobility for CFPS components.

### 2.6 Release of GFP‐His from HAmFU‐DBCO‐Microgels

**Figure 6:**
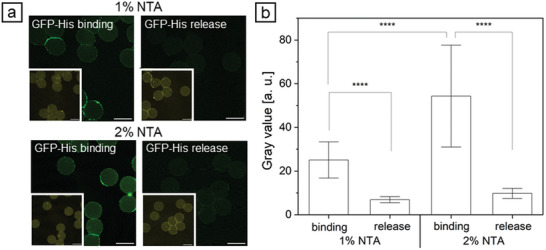
Binding and release of commercial GFP‐His (25 ng µL^−1^ in TRIS buffer) in 1% and 2% NTA‐functionalized HAmFU‐DBCO‐microgels. a) CLSM images of microgels with bound GFP‐His and after release, respectively, inset showing microgels stained with Atto 647N (scale bars indicate 50 µm). b) Calculated gray values of GFP‐His in microgels after binding and release, background was subtracted (n = 50 ± s.d.). Two‐sample *t*‐test, where **** *p* < 0.0001.

Proteins immobilized via Ni^2+^‐activated NTA moieties can be reversibly released by washing with imidazole. To demonstrate the binding capacity of HAmFU‐DBCO‐microgels with 1% and 2% NTA a known concentration of commercial GFP‐His was bound to microgels and subsequently released (**Figure** **6**). It can be seen from the CLSM images that GFP‐His was nearly completely released after successful binding (Figure 6a). In detail, the gray value of HAmFU‐DBCO‐microgels with 1% NTA decreased significantly from 25.1 ± 8.3 when GFP‐His was bound to 6.9 ± 1.4 after release of GFP‐His (Figure 6b). For HAmFU‐DBCO‐microgels with 2% NTA the gray value also declined significantly from 54.3 ± 23.3 for bound GFP‐His to 9.8 ± 2.3 for released GFP‐His. Furthermore, the gray value of bound GFP‐His was significantly higher for 2% NTA than for 1% NTA.

To calculate the binding capacity of GFP‐His in HAmFU‐DBCO‐microgels with 1% NTA or 2% NTA, respectively, the released concentration of GFP‐His in solution was measured and amounted to 3.5 ± 0.3 ng µL^−1^ for 1% NTA (**Table** **2**). For 2% NTA, the released concentration of GFP‐His was 7.2 ± 0.8 ng µL^−1^ and therefore twice as high as for 1% NTA. By determining the microgel concentration with a Neubauer chamber, the amount of GFP‐His per microgel could be calculated, resulting in 0.009 ± 0.002 ng GFP‐His per HAmFU‐DBCO microgel with 1% NTA and 0.014 ± 0.002 ng GFP‐His per HAmFU‐DBCO microgel with 2% NTA.

**Table 2:** Calculation of concentration of released GFP‐His, microgel concentration, and GFP‐His per microgel after binding to HAmFU‐DBCO microgels with 1% NTA and 2% NTA (n = 3 ± s.d.).

	1% NTA	2% NTA
Released concentration of GFP‐His [ng µL^−1^]	3.5 ± 0.3	7.2 ± 0.8
Microgel concentration [µL^−1^]	395 ± 90	510 ± 64
GFP‐His per microgel [ng]	0.009 ± 0.002	0.014 ± 0.002

## 3 Conclusion

Bifunctional microgels were successfully used to covalently attach linear DNA to their porous hydrogel matrix and provide a dense reaction environment for CFPS that allows for the immobilization of in situ synthesized protein. While plasmid DNA is generally more stable and has greater activity in CFPS reaction mixtures than linear DNA, the preparation of linear DNA is faster and less expensive.^[^ ^37^ ^]^ It is ongoing research how CFPS can be adapted for linear DNA and how it can be protected from degradation.^[^ ^40^ ^]^ Here, Chi DNA was successfully utilized to improve CFPS performance. Based on a previous study,^[^ ^31^ ^]^ we expanded the use of bifunctional microgels to tune the amount of protein binding by screening the concentration of available Ni^2+^‐activated NTA moieties presented by the functional hydrogel matrix. Since the same functional groups were used for DNA and NTA binding as well as for crosslinking, the functionalization degree is limited. For HAmFU‐microgels, up to 3% NTA was coupled to microgels, while HAmFU‐DBCO‐DNA‐microgels could be functionalized with up to 2% NTA. For a lower amount of Ni^2+^‐activated NTA moieties at 1% NTA, the in situ synthesized GFP‐His was not quantitatively coupled to microgels but released into the surrounding environment. With a higher amount of Ni^2+^‐activated NTA moieties at 2% NTA, the in situ synthesized GFP‐His was fully coupled to microgels with no protein release in the microgel environment. That way, our bifunctional microgels can serve as a platform to control protein binding and release, thereby regulating protein density inside or outside the microgel. By varying the crosslinker and NTA concentration, the mobility of CFPS components but also synthesized proteins can be tuned. It has to be considered that the diameter of emulsion droplets and microgels, and therefore the DS, vary depending on the functionalization degree, which could influence the assessment of protein binding by CLSM. By combining different linear DNA templates, proteins with and without His‐Tag might be synthesized with tailored immobilization by varying the amount of bound NTA moieties. In this way, dynamic protein crowding in the microgel volume could be achieved to reflect the conditions of macromolecular crowding in vivo more closely. The microgel volume could also serve as a protective environment for DNA and exhibit a crowding effect on its own, which can be further studied by using free DNA instead of bound DNA for CFPS in microgels. After protein synthesis, the microgel network can serve not only for reversible protein immobilization and purification but also for protein stabilization.

## 4 Experimental Section

### Materials

All chemicals were used as received. Device poly(dimethylsiloxane) (PDMS) molding was performed using Sylgard 184 Elastomer Kit (Biesterfeld Spezialchemie GmbH, Hamburg, Germany). Sodium hyaluronate (41 kDa – 65 kDa) was purchased from Lifecore Biomedical (Chaska, MN, USA). 4‐(4,6‐Dimethoxy‐1,3,5‐triazin‐2‐yl)‐4‐methylmorpholinium chloride (DMTMM), *N*,*N*’‐dicyclohexylcarbodiimide (DCC), and 5‐methylfurfurylamine were purchased from TCI (USA). PEG‐mal_2_ (5 kDa) was purchased from JenKem Technology (USA). 1*H*,1*H*,2*H*,2*H*‐perfluoro‐1‐octanol (PFO) was purchased from abcr GmbH (Karlsruhe, Germany). SU‐8 2015 and developer mr‐Dev 600 were obtained from micro resist technology GmbH (Berlin, Germany). FluoroSurfactant was received from RAN Biotechnologies, Inc. (Beverly, MA, USA). 3 M Novec 7500 (HFE 7500) was purchased from IoLiTec‐Ionic Liquids Technologies GmbH (Heilbronn, Germany). Deuterium oxide (D_2_O), deuterated chloroform (CDCl_3_), acetonitrile, sodium sulfate, sodium bicarbonate, 6‐maleimidohexanoic acid, *N*‐hydroxysuccinimide (NHS), *N*_α_,*N*_α_‐Bis(carboxymethyl)‐L‐lysine hydrate, dibenzocyclooctyne‐PEG_4_‐maleimide (DBCO‐PEG_4_‐mal), dimethyl sulfoxide (DMSO), hydrochloric acid, 4‐(2‐hydroxyethyl)‐1‐piperazineethanesulfonic acid (HEPES), magnesium acetate, imidazole, triton X‐100, nickel sulfate (NiSO_4_), folinic acid, t‐RNA, oxalic acid, spermidine, potassium glutamate, and magnesium glutamate were obtained from Sigma‐Aldrich (Merck KGaA, Darmstadt, Germany). Recombinant GFP‐His was purchased from Sino Biological Europe GmbH (Eschborn, Germany). Atto647N‐maleimide was purchased from ATTO‐TEC GmbH (Siegen, Germany). 2‐(*N*‐morpholino) ethanesulfonic acid (MES), tris‐ (hydroxymethyl)aminomethane (TRIS), sodium chloride, potassium acetate, amino acids, nicotinamide adenine dinucleotide, and putrescine were purchased from Carl Roth GmbH (Karlsruhe, Germany). dNTPs were received from New England Biolabs GmbH (Frankfurt, Germany). ATP, UTP, CTP, GTP, and T7 RNA polymerase (T7RNAP) were purchased from Promega GmbH (Walldorf, Germany). Ammonium glutamate and SnakeSkin dialysis tubing (10 kDa MWCO) were purchased from Thermo Fisher Scientific (Waltham, MA, USA). Coenzyme A and 2‐phosphoenolpyruvate (PEP) were purchased from Cayman Chemical (Ann Arbor, MI, USA). *N,N*‐dimethylformamide (DMF) and dichloromethane were obtained from Acros Organics BV (Geel, Belgium). (Tridecafluoro‐1,1,2,2‐tetrahydrooctyl) trichlorosilane was obtained from Gelest, Inc. (Morrisville, PA, USA). ^1^H NMR was performed on a Bruker Avance III. Buffers were prepared in deionized water with a resistance of 18.2 MΩ cm prepared in a Milli‐Q Direct 8 water purification system (Merck Millipore, Burlington, MA, USA). The buffer compositions were as follows:

PBS: 1.7 mm KH_2_PO_4_, 5 mm Na_2_HPO_4_·2H_2_O, 154 mm NaCl, pH 7.4.
TRIS/washing buffer: 20 mm TRIS, 50 mm NaCl, pH 7.2

### Synthesis of HAmFU

According to the synthesis reported by Smith and coworkers,^[^ ^41^ ^]^ 5‐methylfuran‐functionalized hyaluronic acid was prepared with slight modifications.^[^ ^31^ ^]^ Briefly, hyaluronic acid (200 mg, 0.496 mmol) was dissolved in 40 mL of 0.1 m MES buffer (pH 5.5), DMTMM (412 mg, 1.489 mmol) was added to the solution and stirred at RT for 30 min. 5‐Methylfurfurylamine (164 mg, 1.476 mmol) was added dropwise to the solution, and the mixture was stirred for 5 days. Afterward, the reaction mixture was extensively dialyzed (dialysis tubing, 10 kDa MWCO) against aqueous 0.1 m NaCl for 2 days, followed by 2 days against deionized water. Finally, HAmFU was obtained as a white powder by lyophilization and analyzed by ^1^H NMR (500 MHz, D_2_O): δ = 6.34 (s, 1H), 6.07 (d, J = 28.7 Hz, 1H), 4.8‐3.2 (HA backbone), 2.31 (dd, J = 16.5 Hz, 5.9 Hz, 3H), 2.08 ppm (m, 3H). Functionalization degree = 52%.

### Synthesis of HAmFU‐DBCO

The synthesis of HAmFU‐DBCO was performed as previously described.^[^ ^31^ ^]^ First, DBCO‐PEG_4_‐mal (15.38 mg, 0.023 mmol) was dissolved in DMSO. The solution was added dropwise to a solution of HAmFU (100 mg, 0.228 mmol) previously dissolved in 20 mL of Milli‐Q water and allowed to stir for 24 hours. Afterward, the reaction mixture was extensively dialyzed (dialysis tubing, 10kDA MWCO) for 2 days against aqueous 0.1 m NaCl, followed by 2 days against DI. HAmFU‐DBCO was obtained as a slightly yellow powder after lyophilization and analyzed by ^1^H NMR (500 MHz, D_2_O): δ = 7.77 (m, 1H), 7.52 (m, 7H), 6.38 (m, 1H), 6.07 (m, 1H), 5.17 (m, 1H), 4.7‐3.0 (HA backbone/PEG/DBCO), 2.30 (m, 3H), 2.08 ppm (m, 3H).

### Synthesis of NTA‐Maleimide (NTA‐mal)

NTA‐mal was synthesized as described before^[^ ^31^ ^]^ with slight modifications. 6‐Maleimidohexanoic acid (2 g, 9.47 mmol) and NHS (2.18 g, 18.94 mmol) were dissolved in 40 mL of dry DMF under dry and inert conditions. DCC (3.9 g, 18.94 mmol) was added, and the reaction mixture was stirred overnight. To remove excess DCC, oxalic acid (1 g, 0.01 mmol) was added and stirred for another hour. As formed dicyclohexylurea precipitate was filtered off, and DMF was removed by vacuum evaporation. The NHS‐activated ester was dissolved in dichloromethane and extracted with water. Finally, it was dried over Na_2_SO_4_ to give a pale, yellow oil which was analyzed by ^1^H NMR (500 MHz, CDCl_3_): δ = 6.61 (s, 2H), 3.46 (t, J = 7.2 Hz, 2H), 2.76 (m, 4H), 2.53 (t, J = 7.4 Hz, 2H), 1.70 (dt, J = 15.2 Hz,7.5 Hz, 2H), 1.56 (dt, J = 15.0 Hz, 7.5 Hz, 2H), 1.37‐1.31 ppm (m, 2H).

In the next step, the NHS‐activated ester (230 mg, 0.75 mmol) dissolved in 10 mL acetonitrile was added dropwise to a mixture of *N*_α_,*N*_α_‐bis(carboxymethyl)‐L‐lysine hydrate (131 mg, 0.5 mmol) in 10 mL of aqueous 0.3 M NaHCO_3_‐buffer (pH 8.5). After stirring overnight, the solution was acidified to pH 3 with 1 m HCl. The solvents were removed by vacuum evaporation, and the residues were washed with acetonitrile. After drying in vacuo, NTA‐mal was received as white powder, which was analyzed by ^1^H‐NMR (500 MHz, D_2_O): δ = 6.90 (s, 2H), 4.01‐3.93 (m, 5H), 3.57 (t, J = 6.9 Hz, 2H), 3.25 (m, 2H), 2.28 (t, J = 7.2, 2H), 2.04‐1.93 (m, 2H), 1.69‐1.55 (m, 8H), 1.35‐1.29 ppm (m, 2H).

### Microfluidic Device Fabrication

PDMS‐based microfluidic flow‐focusing devices were prepared by combined photo‐ and soft lithography.^[^ ^42^ ^]^ Briefly, a negative photoresist SU‐8 2015 was spin‐coated onto a 3‐in. silicon wafer (SIEGERT WAFER GmbH, Aachen, Germany). Using a mask aligner (MJB3, SÜSS MicroTec SE, Garching, Germany), computer‐aided channel design was transferred from a photomask onto the photoresist. Non‐polymerized photoresist was removed by washing with developer solution mr‐Dev 600. Subsequently, a 10:1 ratio of PDMS oligomer and crosslinker was mixed and degassed in a planetary centrifugal mixer (ARE‐250, Thinky, Laguna Hills, CA, USA), poured onto the master structure, and cured for 2 h at 80 °C. In‐ and outflow ports were punched into the PDMS replica, and the device was bonded to a glass slide by oxygen plasma treatment (80 W for 60 s, MiniFlecto 10, Plasma Technology, Herrenberg, Germany). Prior to usage, the microchannels were treated with a solution of 1% (v/v) trichloro(1*H*,1*H*,2*H*,2*H*‐perfluorooctyl)silane in HFE 7500 to increase the hydrophobicity of the microchannel walls.

### Microfluidic Preparation of NTA‐Functionalized HAmFU‐PEG‐mal_2_ Microgels

A batch of NTA‐functionalized microgels was composed of 3.5% (w/v) HAmFU, 1–4% (w/v) NTA‐mal, 1% (w/v) PEG‐mal_2_, and 0.3 µL Atto647N‐maleimide (1 µg µL^−1^) dissolved in PBS buffer. HAmFU was pre‐incubated with NTA‐mal for 1 h and Atto647N‐maleimide for 30 min at 600 rpm, respectively. Emulsion droplets were formed in a microfluidic flow‐focusing device with a channel height and a junction width of 25 µm each, which was connected to high‐precision syringe pumps (Pump 11 Pico Plus Elite, Harvard Apparatus, MA, USA) via PE tubing (inner diameter: 0.38 mm; outer diameter: 1.09 mm, Hartenstein, Würzburg, Germany). 500 µL gastight syringes (1750 TLL SYR, Hamilton, Reno, NV, USA) were used for the co‐injection of dispersed phases (DP) containing solutions of PEG‐mal_2_ and NTA‐functionalized HAmFU, respectively. A 3 mL disposable syringe (BD Luer lock tip, Becton Dickinson, Franklin Lakes, NJ, USA) was used for the continuous phase (CP), which is composed of HFE 7500 supplemented with 2% (w/w) of surfactant. The flow rates were set to 500 µL h^−1^ for the CP, 50 µL h^−1^ for the DP containing PEG‐mal_2_, and 20 µL h^−1^ for the DP with NTA‐pre‐incubated HAmFU solution. Microfluidic droplet formation was followed on an Axio Vert.A1 (Carl Zeiss Microscopy GmbH, Germany) equipped with a Phantom Miro C110 high–speed digital camera (Vision Research Inc., USA). Microgel gelation was completed overnight at RT. For purification, microgels were transferred into the respective buffer by performing three washing steps with 20% (v/v) PFO in HFE 7500, and removing the oil phase by centrifugation at 0.6 rcf for 30 s. An additional washing step was performed with 0.5% (v/v) triton in PBS, followed by consecutively washing with MilliQ water.

### Protein Immobilization and Release Inside NTA‐Functionalized Microgels

For protein immobilization studies, NTA‐functionalized HAmFU‐ or HAmFU‐DBCO‐microgels were transferred into an aqueous NiSO_4_ (750 mM) solution and incubated for 1 h at RT at 900 rpm. Afterward, microgels were washed six times with TRIS buffer to remove excess Ni^2+^‐ions. 180 µL of microgels were incubated with 20 µL of GFP‐His (0.25 µg µL^−1^) for 1 h at RT and 900 rpm. Upon incubation, microgels were washed three times with TRIS buffer and one time with buffer containing 20 mm TRIS, 50 mm NaCl, and 10 mm imidazole (pH 7.2). For elution of immobilized GFP‐His, the microgels were transferred to 20 mm TRIS, 50 mm NaCl, and 250 mm imidazole (pH 7.2), and incubated for 1 h at RT and 900 rpm. For observation with CLSM (Leica TCS SP8, Leica Microsystems GmbH, Germany) microgels were transferred into PDMS chambers with a height of 50 µm.

### Plasmid pET28a‐deGFP‐His Isolation

For plasmid isolation *E. coli* TG1 cells transformed with pET28a‐deGFP‐His were used. The sequence of deGFP was derived from pBEST‐OR2‐OR1‐Pr‐UTR1‐deGFP‐T500 (Addgene plasmid #40019)^[^ ^15^ ^]^ and subcloned into pET28a(+) DNA‐Novagen vector (Merck KGaA) to introduce a His‐Tag.^[^ ^31^ ^]^ The plasmid was isolated with ZymoPURE II Plasmid Midiprep Kit (Zymo Research Europe GmbH, Germany). Afterward, DNA purity and concentration were checked by electrophoresis with 1% agarose gel and on a NanoDrop ND‐1000 spectrophotometer (Thermo Fisher Scientific, Germany).

### PCR Reaction

PCR reactions were performed on a thermocycler FlexCycler^2^ (Analytik Jena AG, Germany) in 50 µL reaction volumes using 1.25 U Taq polymerase with ThermoPol buffer (New England BioLabs), 200 µm dNTPs, 0.2 µm forward primer, 0.2 µm reverse primer (cf. Table 4), and 100 ng µL^−1^ of the isolated plasmid pET28a‐deGFP‐His. The reaction was incubated for 3 min at 95 °C, followed by 35 cycles of 30 sec each at 95 °C, 45 sec at 56 °C, and 2 min at 68 °C. The final extension step was conducted for 5 min at 68 °C, and the reaction mixture was stored at 4 °C. Purification was performed using a GeneJET PCR Purification Kit (Thermo Fisher Scientific, Germany). DNA purity and concentration were checked as described above.

### CFPS in Solution

CFPS was performed by comparing two different approaches. The RTS 100 E. coli HY Kit (biotechrabbit GmbH, Berlin, Germany) was used by following the instructions provided by the manufacturer and using 5 nm PCR product or 5 nm plasmid DNA as a DNA template and no DNA as control. In another approach, a CFPS reaction was prepared according to Levine et al.^[^ ^14^ ^]^

After preparation of solution A and B (**Table** **3**) they were mixed with 44 U of T7‐RNAP, 5 nm PCR product or 5 nm plasmid DNA as DNA template, optional 2 µm Chi6 DNA (**Table** **4**),^[^ ^38^ ^]^ and 33% (v/v) *E. coli* lysate HighYield‐T7 (Cube Biotech GmbH, Monheim, Germany). The fluorescence of the synthesized GFP‐His was measured every 5 minutes, with an excitation wavelength of 488 nm and an emission wavelength of 520 nm using a Tecan Infinite M200 PRO plate reader (Tecan Trading AG, Switzerland).

**Table 3:** Composition of CFPS reaction mixture consisting of solution A and B that were separately prepared as stock solutions according to Levine et al.^[^ ^14^ ^]^ and combined afterward to give the final concentrations as listed below.

Solution A Reagent	Concentration
Folinic acid	0.09 mm
UTP, CTP, GTP	each 1.28 mm
ATP	1.81 mm
NAD	0.4 mm
coenzyme A	0.25 mm
oxalic acid	0.82 mm
spermidine	1.48 mm
putrescine	0.55 mm
t‐RNA	0.26 mg mL^−1^
HEPES	58.80 mm

Solution B Reagent	Concentration
K‐glutamate	130.21 mm
NH‐glutamate	8.12 mm
Mg‐glutamate	9.97 mm
PEP	33.08 mm
Amino acids	each 2.0 mm

**Table 4:** DNA primer for PCR and Chi DNA added in CFPS. All strands were purchased from biomers.net GmbH (Germany). Sequence of Chi6 DNA was related to Marshall et al.^[^ ^38^ ^]^

DNA	Modification	Sequence (5′ → 3′)
Forward primer	Atto565	GAG CCC GAT CTT CCC CAT CG
Reverse primer	Azide‐PEG_4_	GCG TAA CCA CCA CAC CCG
Forward Chi6	‐	TCACTTCACTGCTGGTGGCCACTGCTGGTGGCCACTGCTGGTGGCCACTGCTGGTGGCCACTGCTGGTGGCCACTGCTGGTGGCCA
Reverse Chi6	‐	TGGCCACCAGCAGTGGCCACCAGCAGTGGCCACCAGCAGTGGCCACCAGCAGTGGCCACCAGCAGTGGCCACCAGCAGTGAAGTGA

### Microfluidic Fabrication of DNA/NTA‐Modified HAmFU‐DBCO‐PEG‐mal_2_ Microgels

3.7% (w/v) HAmFU‐DBCO was dissolved in PBS buffer and subsequently incubated with 90 nm azide‐modified PCR product for 20 h. Afterward, NTA‐mal was added to the solution and incubated for 1 h at RT at 600 rpm. Then, 0.3 µL of Atto647N‐maleimide (1 µg µL^−1^) was added and incubated at RT for 30 minutes. Prior to injection into the flow‐focusing microfluidic device with a channel height and width of 25 µm, the solution was mixed with 0.5% (w/v) PEG‐mal_2_. The W/O emulsion droplets were formed with flow rates of 50 µL h^−1^ for DP and 500 µL h^−1^ for CP. Microgel gelation was completed for 48 h at RT. For purification, microgels were transferred into the respective buffer by performing three washing steps with 20% (v/v) PFO in HFE 7500 and removing the oil phase by centrifugation at 0.6 rcf for 30 s. DNA/NTA‐modified microgels were washed thrice with Milli‐Q water to remove non‐covalently bound DNA.

### Ribosome Mobility

NTA‐modified HAmFU‐DBCO microgels were transferred into physiological buffer (25 mM HEPES, 50 mm potassium glutamate, 50 mm potassium acetate, 5 mm magnesium acetate) and incubated with 1 µm *E. coli* ribosomes labeled with Atto‐488, which were prepared es described before.^[^ ^43^ ^]^ After incubation over night at 4 °C the microgels were transferred into PDMS chambers with a height of 50 µm for observation with CLSM (Leica TCS SP8 Leica Microsystems GmbH, Germany).

### CFPS and Protein Immobilization in Microgels

DNA‐/NTA‐modified HAmFU‐DBCO‐microgels were transferred into an aqueous 0.01 m NiSO_4_ solution and incubated for at least 20 h at RT at 900 rpm. Afterward, Ni^2+^‐activated DNA‐/NTA‐microgels were washed six times with MilliQ water to remove excess Ni^2+^ ions and combined with the CFPS reaction mixture with the same concentration as used before but in a reduced volume of 10 µL to fit into observing chambers (Grace Bio‐Labs SecureSeal (Sigma‐Aldrich, Germany)). The volume of the PCR product, which was used for CFPS in solution, was replaced by DNA‐/NTA‐microgels. The reaction mixture was placed into observing chambers between two glass slides, and the CFPS was observed over time via CLSM (Leica TCS SP8, Leica Microsystems GmbH, Germany).

### Data Analysis

Diameters of emulsion droplets and microgels as well as gray values after GFP‐His immobilization, CFPS of GFP‐His and ribosome mobility were measured manually using ImageJ.^[^ ^44^ ^]^ Data analysis and statistical calculations (two‐sample *t*‐test) were carried out using Origin and GraphPad.

## Conflict of Interest

The authors declare no conflict of interest.

## Author Contributions

A.K. conceptualized the idea for the study; performed data curation, formal analysis, investigation, validation, and visualization; wrote the original draft; and wrote, reviewed, and edited the manuscript. K.I. performed data curation, formal analysis, investigation, and validation; visualized the idea for the study; and wrote the original draft. J.T. conceptualized the idea for the study; performed funding acquisition; and wrote, reviewed, and edited the manuscript.

## Supporting information



Supporting Information

## Data Availability

The data that support the findings of this study are available from the corresponding author upon reasonable request.
