# Supplementary material for: Regulating Protein Immobilization During Cell‐Free Protein Synthesis in Hyaluronan Microgels
Source: Adv Biol (Weinh). 2025 Feb 17;9(5):2400668. doi: 10.1002/adbi.202400668 (PMC12078891; doi:10.1002/adbi.202400668)
Supplement: Supplementary file 1 — Supporting Information [file ADBI-9-2400668-s001.docx]

**Regulating protein immobilization during cell-free protein synthesis in hyaluronan microgels**

*Anika Kaufmann,*^1,*^ *Kateryna Ivanova,^1^ Julian Thiele^1,2^*

^1^ Leibniz-Institut für Polymerforschung Dresden e.V., 01069 Dresden, Germany

^2^ Institute of Chemistry, Otto von Guericke University Magdeburg, 39106 Magdeburg, Germany

* Email: [kaufmann@ipfdd.de](mailto:kaufmann@ipfdd.de)

**Supporting information**


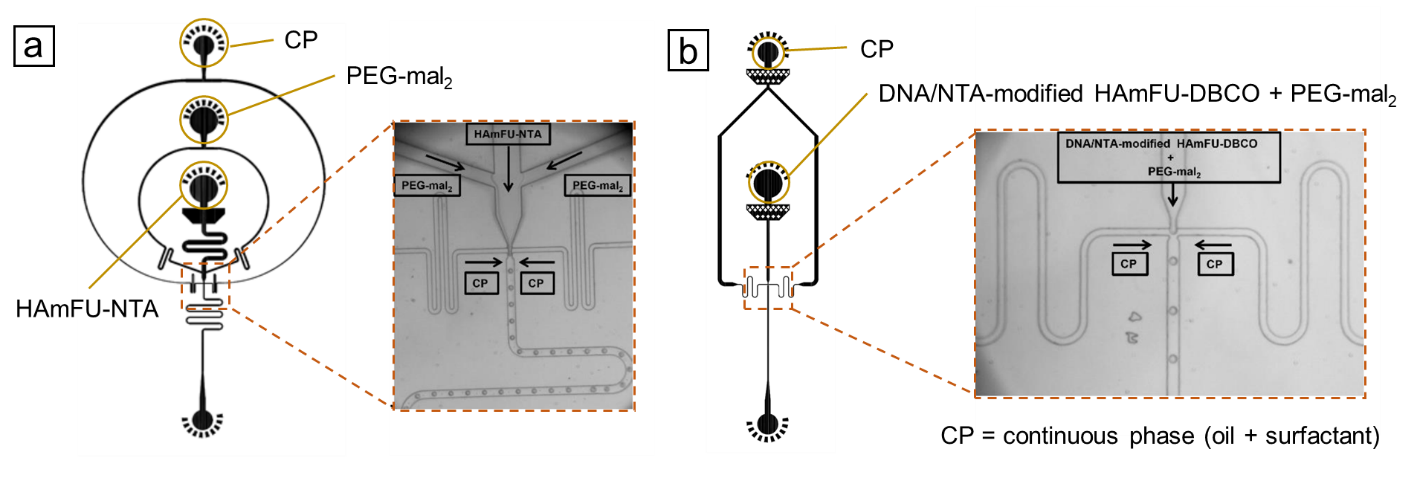


Figure S1. Scheme of the microfluidic devices and their corresponding brightfield microscopy images of the flow-focusing junction used for the fabrication of (a) NTA-functionalized HAmFU-microgels and (b) DNA/NTA-modified HAmFU-DBCO-microgels.


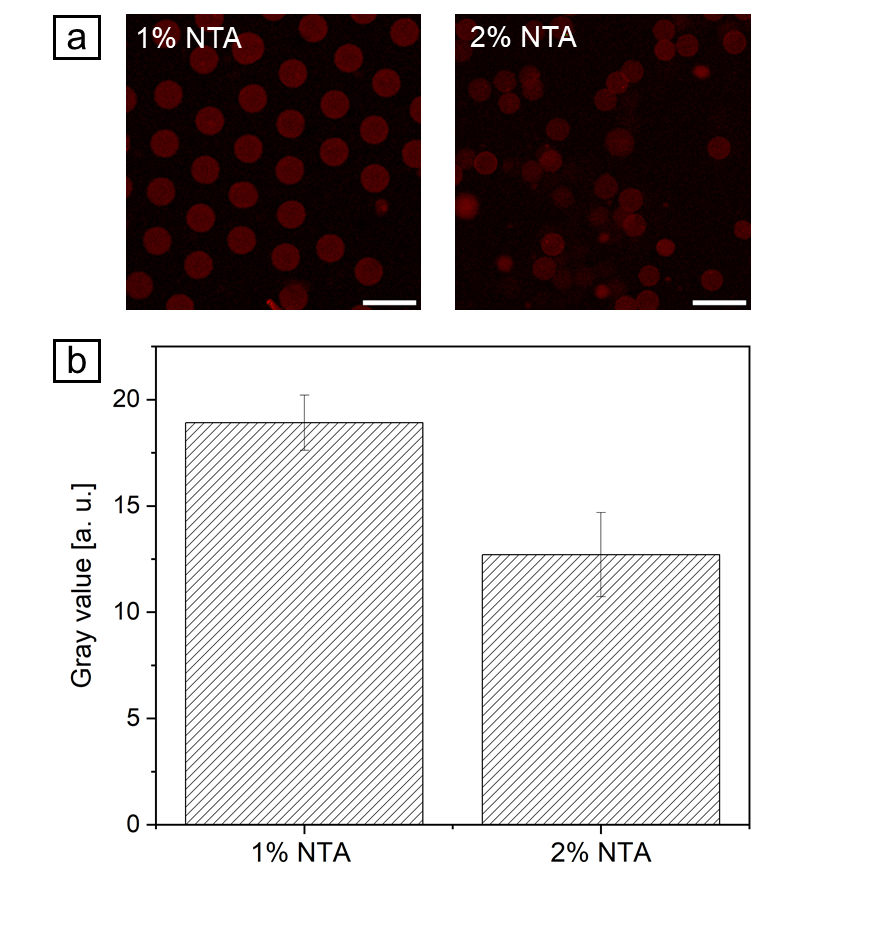


**Figure S2.** Analysis of DNA bound to 1% and 2% NTA containing HAmFU-DBCO-DNA-microgels. (a) CLSM images after 20 h, (b) corresponding calculated gray values, background was subtracted (n = 50 ± s.d.).


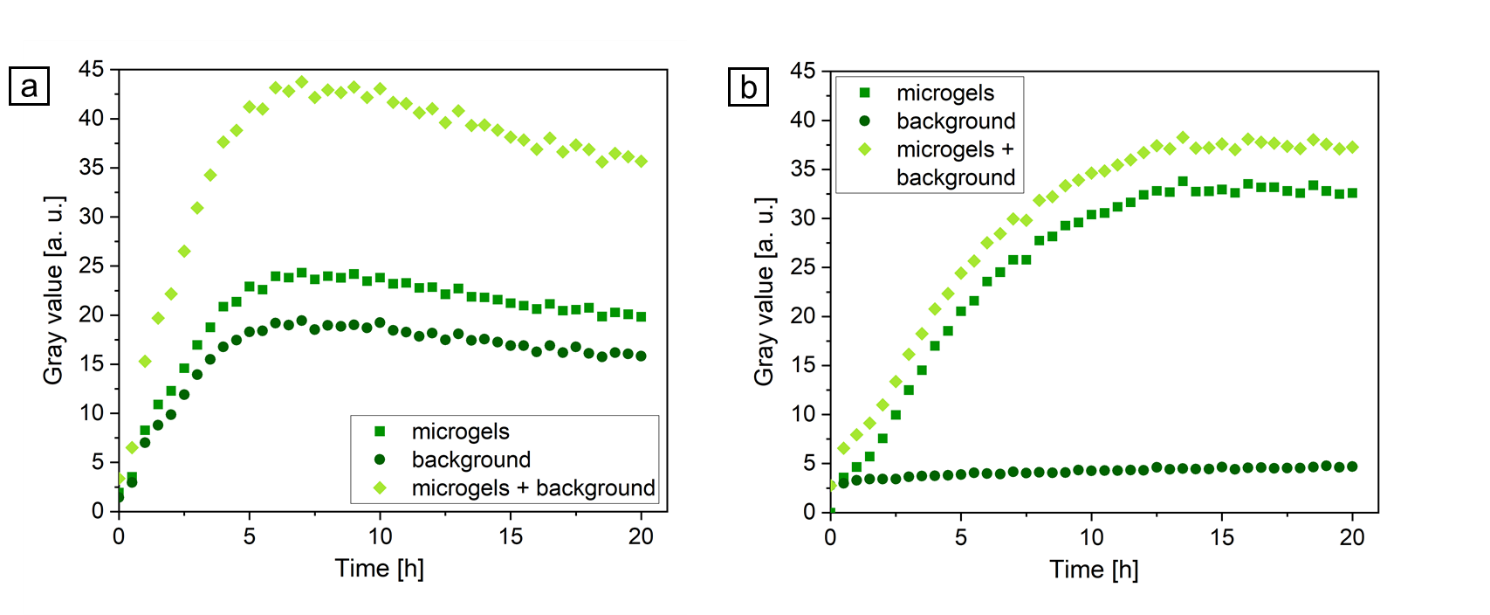


**Figure S3.** Time-dependent change in gray values of GFP-His after CFPS in HAmFU-DBCO-DNA-microgels functionalized with (a) 1% NTA and (b) 2% NTA.
